# Amphiphilic Lipopeptide-Mediated Transport of Insulin and Cell Membrane Penetration Mechanism

**DOI:** 10.3390/molecules201219771

**Published:** 2015-12-03

**Authors:** Yu Zhang, Lei Li, Mei Han, Jiaoyin Hu, Liefeng Zhang

**Affiliations:** Jiangsu Key Laboratory for Molecular and Medical Biotechnology, College of Life Science, Nanjing Normal University, Nanjing, 210046, China; zhangyunjnu@163.com (Y.Z.); mcu430@163.com (L.L.); mmmmhanmei@163.com (M.H.); 08253@njnu.edu.cn (J.H.)

**Keywords:** amphiphilic lipopeptide, insulin, membrane penetrating action

## Abstract

Arginine octamer (R8) and its derivatives were developed in this study for the enhanced mucosal permeation of insulin. R8 was substituted with different aminos, then modified with stearic acid (SA). We found that the SAR6EW-insulin complex had stronger intermolecular interactions and higher complex stability. The amphiphilic lipopeptide (SAR6EW) was significantly more efficient for the permeation of insulin than R8 and R6EW both *in vitro* and *in vivo*. Interestingly, different cellular internalization mechanisms were observed for the complexes. When the effectiveness of the complexes in delivering insulin *in vivo* was examined, it was found that the SAR6EW-insulin complex provided a significant and sustained (six hours) reduction in the blood glucose levels of diabetic rats. The improved absorption could be the comprehensive result of stronger intermolecular interactions, better enzymatic stability, altered internalization pathways, and increased transportation efficacy. In addition, no sign of toxicity was observed after consecutive administrations of SAR6EW. These results demonstrate that SAR6EW is a promising epithelium permeation enhancer for insulin and suggest that the chemical modification of cell-penetrating peptides is a feasible strategy to enhance their potential.

## 1. Introduction

Insulin is the most effective and durable drug in the treatment of advanced-stage diabetes. Despite significant advancements in pharmaceutical research, the development of a proper non-invasive insulin delivery system remains a major challenge [1]. Oral delivery is a convenient and patient-friendly route of drug administration compared to injections, however, the oral delivery of insulin has shown limited therapeutic efficacy [2,3]. There are two main limitations of the oral route for insulin delivery. One is insulin degradation by gastrointestinal proteolytic enzymes. The other is the poor permeability of insulin across the intestinal epithelium due to its high molecular weight and lack of lipophilicity [4]. Therefore, it is necessary to develop a safe and efficient oral delivery system for insulin.

Different formulation approaches have been investigated to overcome the gastrointestinal tract barriers for the delivery of insulin via the oral route, such as the use of liposomes, microemulsions, microspheres, and nanoparticles [5]. Cell-penetrating peptides (CPPs) have attracted increasing attentions in recent years as a promising vehicle for facilitating the cellular uptake of various molecular cargos [6]. CPPs facilitate the transduction of macromolecules such as proteins [7], liposomes [8,9], and antibodies [10] into cells. Previous studies have shown that CPPs can enhance intestinal insulin absorption by co-administration as a physical mixture without intermolecular cross-linking [11,12,13]. Among several types of CPPs, the arginine-rich CPPs have been the most widely studied [14,15]. Oligoarginine, a cell-penetrating peptide, significantly enhances intestinal insulin absorption without any untoward effects on the intestinal mucosa [12,16].

Structure-activity studies are very important for CPPs. There are a few examples in the literature where alterations in the chemical/structural parameters of the peptide have been found to be crucial for improving its cargo delivery efficiency [17]. For example, structural rearrangements and chemical modifications in known cell penetrating peptides strongly enhance DNA delivery efficiency [18]. Therefore, knowing the structure of CPPs is important to better facilitate the entry of these foreign molecules across the plasma membrane. Furthermore, stearylation of CPPs has proven to be another successful methodology to increase the transfection efficiency of both plasmids [19] and siRNA [20], through a non-covalent approach. Improved efficiency in CPP-mediated delivery by lipidation has also been reported [21]. However, only limited information is available about the intracellular fate of the chemically modified oligoarginines and their applicability as a delivery vector for peptides/proteins.

In the present study, we have used R8 as a model peptide system and systematically changed its structure by substituting with different aminos, then modifying with fatty acids having various chain lengths. Firstly, we found stearyl (C18) had stronger binding to insulin than decanoyl (C10) and tetradecanoyl (C14) using computer simulation methods. The (SAR6EW-insulin) complex was prepared in order to improve the stability and bioavailability of orally administered insulin. The effectiveness of the complex for intestinal delivery of insulin was evaluated both *in vitro* and *in vivo*. Hypoglycemic activity was evaluated on diabetic rats. However, the mechanism by which these CPPs translocate across cell membranes is still not clearly understood. Herein, the mechanism of improving permeation enhanced by SAR6EW was investigated in terms of intermolecular interaction, cellular uptake, and specific inhibition studies.

## 2. Results and Discussion

### 2.1. Circular Dichroism Spectroscopy

**Figure 1:**
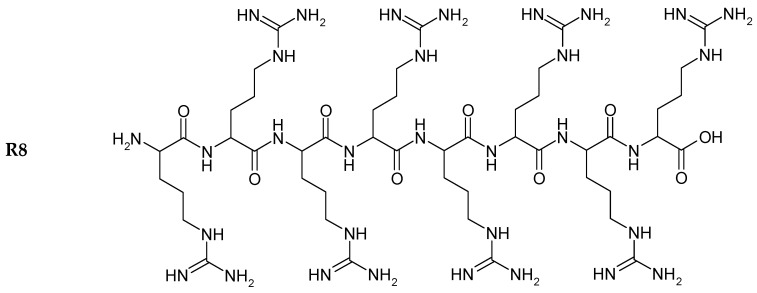

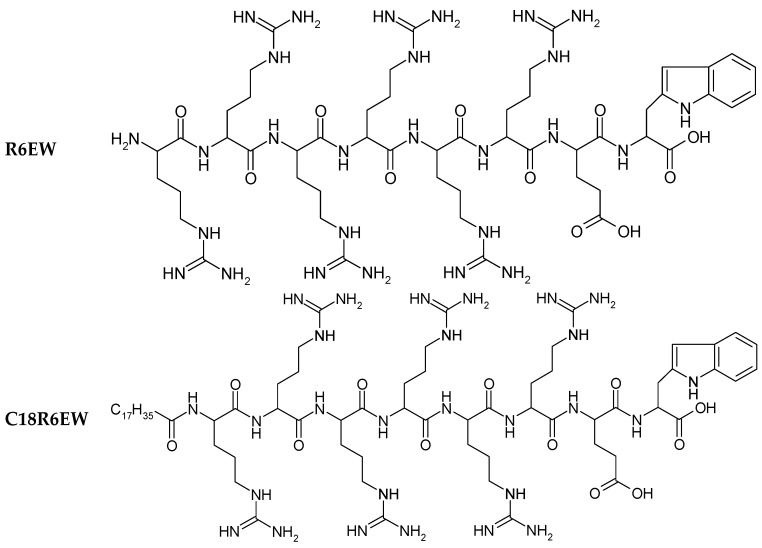
Chemical structure of peptides.

**Figure 2:**
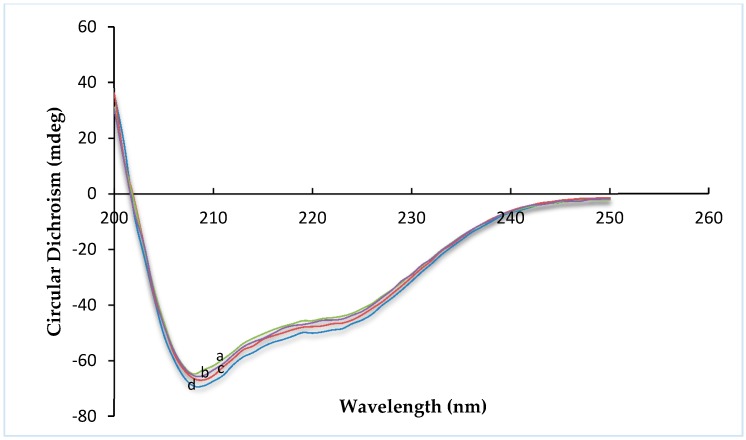
Far-UV CD spectra. a, insulin; b, R6EW-insulin; c, R8-insulin; d, SAR6EW-insulin.

Circular dichroism (CD) is an excellent tool for investigating the secondary structure of proteins in solution. The far-UV range of the CD spectra provides information about α-helix and β-sheet conformation on various protein structures [22]. The structures of R8 and the derivatives are shown in Figure 1. Insulin exhibited the typical spectrum of an α-helical protein, with characteristic minima at 208 and 222 nm. Far-UV-CD spectra (Figure 2) were recorded in the range of 200–250 nm, at 25 °C. Any change in the secondary structure of insulin would be reflected as variations in the spectral intensity in the vicinity of these peaks. No significant difference was found between insulin, R8-insulin, R6EW-insulin and SAR6EW-insulin. Regardless of a slight difference in the spectral intensity and fractional composition of secondary parameters, the spectral shape of the complexes was similar to that of native insulin. To sum up, R8, R6EW and SAR6EW did not alter the CD spectra. This suggests that the binding does not alter the secondary structure of insulin.

### 2.2. Isothermal Titration Calorimetry (ITC)

**Figure 3:**
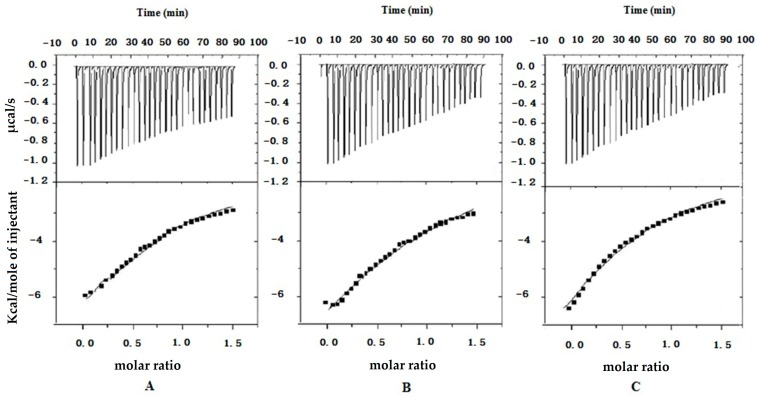
ITC data from the titration of R8 (**A**); R6EW (**B**) and SAR6EW (**C**) in the presence of insulin. In the top panel, the peaks indicate the heat released after each addition. The peaks were integrated and the total heat released per injection (peak area) is plotted as a function of molar ratio in the bottom panel.

ITC is becoming the method of choice for characterizing intermolecular interactions and recognizing reactions with exquisite sensitivity. Figure 3 shows the ITC curves of the binding of insulin to R8 (A), R6EW (B) and SAR6EW (C) at 25 °C. Typical ITC titration curves corresponding to the binding interaction are presented. It was observed that the binding of insulin to R8 derivatives was exothermic. Since the ITC measurements of the binding were obtained under identical conditions, a direct comparison of the thermodynamic parameters among them was possible. The results were the following. The three fitting parameters, stoichiometry (N), enthalpy (ΔH) and binding constant (K) are shown in Table 1.

**Table 1:** The three fitting parameters: stoichiometry (N), enthalpy (ΔH) and binding constant (K).

Formulations	ΔH (Kcal/mol)	K (M^−1^)	N
R8 to insulin	−9.2 ± 0.365	2.1 ± 0.2 × 10^4^	0.32 ± 0.4
R6EW to insulin	−10.4 ± 0.324	2.7 ± 0.2 × 10^4^	0.46 ± 0.5
C18R6EW to insulin	−14.8 ± 0.458	4.6 ± 0.3 × 10^4^	0.78 ± 0.6

The ΔH value of SAR6EW to insulin is the lowest, indicating that the binding of SAR6EW is more exothermic than those of R8 and R6EW. The K value of SAR6EW to insulin is the highest, demonstrating that SAR6EW binds more tightly to insulin than the others. The binding strength was therefore in this order: SAR6EW-insulin > R6EW-insulin > R8-insulin. The results showed R6EW had more binding to insulin than R8. It may be due to the different amino acid compositions. Furthermore, SAR6EW had the strongest binding affinity to insulin among the CPPs. N-terminal stearylation of the R6EW (SAR6EW), which has an amphiphilic structure, may contribute to the increased the intermolecular interaction. Electrostatic and hydrophobic interactions might exist between SAR6EW and insulin, while only electrostatic interaction was present between R8 (R6EW) and insulin.

### 2.3. Enzymatic Degradation Study

Protection of insulin against proteolytic enzymes was investigated *in vitro* by incubating the obtained oligoarginine derivative/insulin in the presence of enzymes such as trypsin or α-chymotrypsin, the major intestinal tract proteases [23].

**Figure 4:**
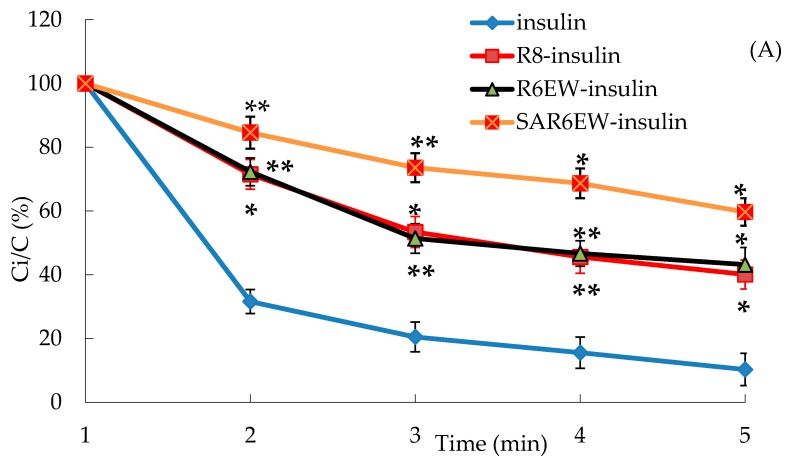

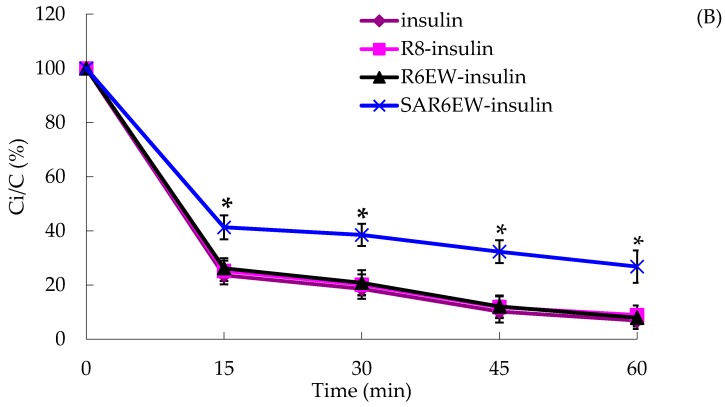
(**A**) Percentage of insulin remaining in the presence of trypsin at 37 °C; (**B**) Percentage of insulin remaining in the presence of α-chymotrypsin at 37 °C (mean ± SD, N = 3, *: *p* < 0.05, **: *p* < 0.01 *vs.* control). Ci/C (%) means the percentage of insulin remaining.

In the presence of trypsin, the amount of insulin decreased with incubation time from 0 to 60 min, and only about 15% of insulin remained after 60 min. The degradation of insulin was partly inhibited in the presence of R8 and R6EW, and was significantly decreased when incubated in the presence of SAR6EW (Figure 4A). On the other hand, insulin was degraded rapidly by α-chymotrypsin, and most of the insulin was degraded within 60 min in the insulin, R8-insulin and R6EW-insulin. However, about 32% of insulin was detected in the presence of SAR6EW.

Results from the degradation by α-chymotrypsin demonstrated that there were no significant differences among insulin, R8-insulin and R6EW-insulin. SAR6EW had an advantage on the protection of insulin. Simultaneously, we found CPPs (R8 and R6EW) were useful in resisting degradation by trypsin, but the effect of SAR6EW was more pronounced. Viewed together, SAR6EW had an obvious effect on protecting insulin from degradation by the intestinal proteases. In addition, the increase in hydrophobic interactions might result in improving the stability of the complex.

### 2.4. In Vitro Cellular Uptake and Transport Studies

Intracellular uptakes of insulin, R8-insulin, R6EW-insulin and C18R6EW-insulin were evaluated on the Caco-2 cell model, which is derived from a human colorectal carcinoma, and show close morphological and functional similarities with intestinal epithelium [24].

**Figure 5:**
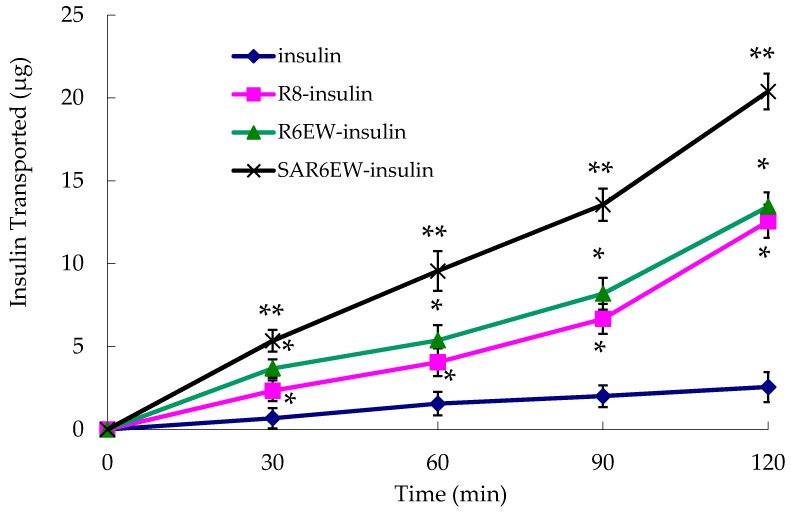
Amount of insulin transported across Caco-2 cell monolayer during the permeation experiments (mean ± SD, N = 3, *: *p* < 0.05, **: *p* < 0.01 *vs.* control).

The experiment was performed to investigate the effects of R8 derivatives on the transport properties of insulin across the Caco-2 cell monolayer. As shown in Figure 5, the transportation efficacy of native insulin crossing the Caco-2 cell monolayer was extremely low. However, R8 and R6EW, to some extent, caused an increase in the transport of insulin across the monolayer. Higher transport efficiency was obtained with R6EW-insulin than R8-insulin. The presence of SAR6EW significantly enhanced the transport efficiency. Transport across the Caco-2 cell monolayer was in this order: SAR6EW-insulin > R6EW-insulin > R8-insulin > insulin. In this study, we found that SAR6EW showed the highest increase in the permeability of insulin across the Caco-2 cell monolayer of all the formulations studied. It was reported that acylation of octaarginine enhancing cellular uptake was attributed to the increased interaction between the vector and membranes [25]. However, only studying the affinity to plasma membranes is not enough to elucidate the mechanism. It was also found that the same vector with different proteins had different uptake mechanisms [26]. It suggested that the increasing efficiency was involved with not only the affinity of vector to plasma membranes, but also the binding between vector and drugs.

### 2.5. Mechanism of Cellular Uptake

**Figure 6:**
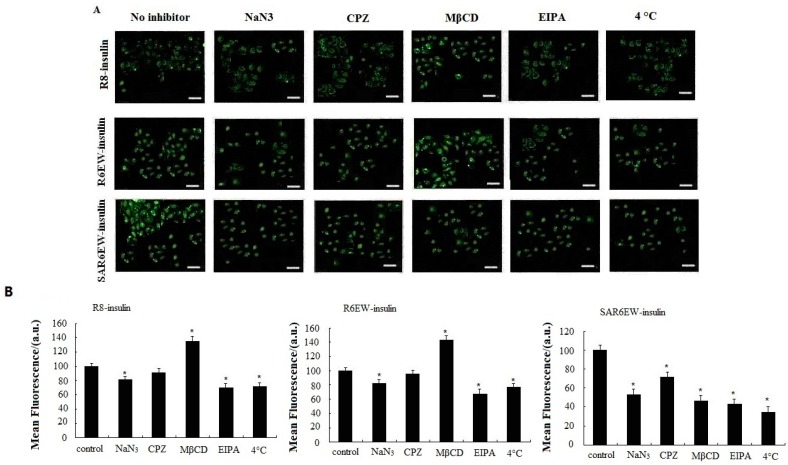
Caco-2 cellular uptake of F-insulin in the presence of various endocytosis inhibitors or at different temperatures analyzed by CLSM (**A**) and flow cytometry (**B**). *: *p* < 0.05.

To investigate the uptake mechanism of the insulin formulations, specific inhibition studies were performed. Sodium azide (NaN_3_) was used for energy depletion as a comprehensive active transport inhibitor. Chlorpromazine (CPZ), methyl-β-cyclodextrin (MβCD), and 5-(*N*-ethyl-*N*-isopropyl) amiloride (EIPA) were used as specific endocytosis inhibitors for clathrin-mediated endocytosis, caveloae/lipid-raft-mediated endocytosis, and macropinocytosis, respectively [27]. The effects of all inhibitors on the delivery of R8-insulin, R6EW-insulin and SAR6EW-insulin were tested. Results are shown in Figure 6. For R8-F-insulin and R6EW-F-insulin, CPZ presented no effect on the uptake, and NaN_3_ and EIPA could decrease the fluorescent signals, but MβCD increased them. However, for SAR6EW-F-insulin, the cellular uptake was inhibited in the presence of NaN_3_, MβCD, CPZ and EIPA. For insulin little signal was detected by CLSM and flow cytometry (figure not shown). This result suggested that different internalization pathways might exist. Since energy depletion has effects on the internalization of R8-insulin, R6EW-insulin and SAR6EW-insulin, their uptake might occur via energy-dependent pathways. However, different modes of endocytosis were involved.

Endocytosis is an energy-dependent pathway and is usually suppressed when the cells are treated at 4 °C [28]. A temperature-dependent uptake study was performed at 37 °C and 4 °C to further verify the uptake mechanism. A significant decrease in the fluorescent intensity was observed when the cells were treated with R8-F-insulin, R6EW-F-insulin and SAR6EW-F-insulin at 4 °C. However, they still yielded fluorescent signals. These results suggested the possibility that both an energy dependent pathway route and direct penetration were involved. Simultaneously, the results of all inhibitors’ effects analyzed by CLSM and flow cytometry were consistent.

Although numerous studies about the uptake mechanism of CPPs across the plasma membranes have been reported, the pathways through which CPPs enter the cells have not been clearly described [29]. The mechanisms can be categorized into energy-dependent endocytosis and energy-independent direct transduction across the lipid bilayer [30]. In this mechanistic study, it was suggested that both an energy-dependent pathway route and energy-independent pathways (direct transduction) were involved in the three complexes. However, different modes of endocytosis were observed. Interestingly, MβCD even increased the uptake of R8-insulin and R6EW-insulin. This phenomenon was also observed by other researchers, and they considered that perturbation of endocytotic mechanisms promoted the accumulation of peptides on the membrane for cellular import via direct transduction [31]. Previous studies have proved that CPPs can harness alternate mechanisms of uptake according to the environmental conditions where experimental conditions differ in some important aspects [32]. Therefore, we studied the mechanism under the same conditions in order to eliminate the variables. The difference in uptake mechanisms may be due to the variation of the intermolecular interactions and complex stabilities.

### 2.6. Insulin Delivery in Diabetic Rats

**Figure 7:**
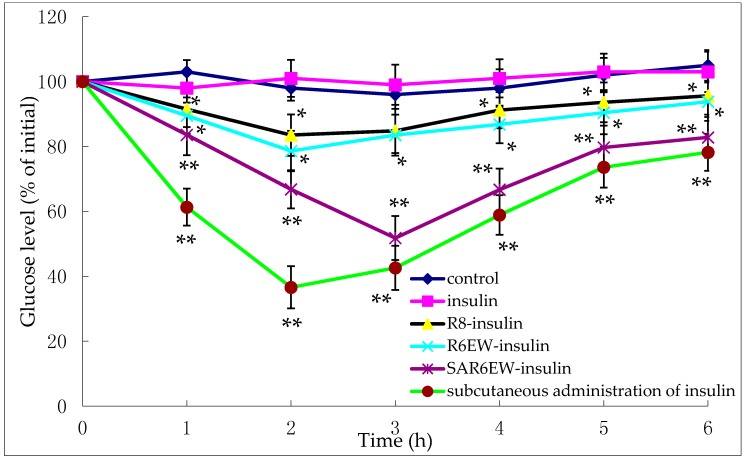
Plasma glucose levels (% of initial) after oral administration of the following compounds in diabetic rats (mean ± SD, *n* = 3, *: *p* < 0.05, **: *p* < 0.01 *vs.* control).

The pharmacological effects were evaluated on diabetic rats. Figure 7 shows the variation of blood glucose concentrations after their intestinal administration (25 IU/kg) or subcutaneous injection (1 IU/kg) of insulin solution. 

These findings show the glycemic variation in diabetic rats expressed as a percentage of the animal’s initial glycemia after oral administration of either an insulin-containing formulation or control. No reduction in glycemia was observed after the intestinal administration of insulin solution and control. This demonstrated the stable diabetic level in the experimental animals. In contrast, a reduction in glycemia was observed after the administration of R8-insulin and R6EW-insulin. From statistical analysis, this finding had statistical significance (*p* < 0.05). Glycemia decreased more following the administration of R6EW-insulin than R8-insulin. A marked reduction in glycemia was also observed with SAR6EW-insulin. It exhibited the strongest hypoglycemic effect with a maximum reduction of 51%. The blood glucose level was maintained at a low level for up to 6 h. The improved absorption could be the comprehensive result of stronger intermolecular interactions, better enzymatic stability, altered internalization pathways, and increased transportation efficacy. Moreover, these factors might affect each other. 

### 2.7. Toxicity Study

**Figure 8:**
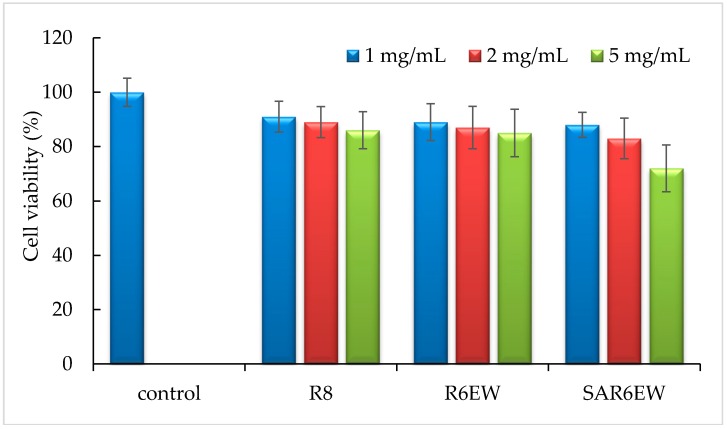
Cytotoxicity of R8 derivatives on Caco-2 cells at 1, 2, and 5 mg/mL (mean ± SD, *n* = 3).

Cytotoxicity was evaluated at the concentrations of 1, 2, and 5 mg/mL. As shown in Figure 8, the relative cell viability from 1–5 mg/mL had no significant difference compared with the control. At 5 mg/mL, a reduction in cell viability was observed for SAR6EW. From the statistical analysis, this finding had no statistical significance (*p* > 0.05). These results showed no significant difference compared with the control.

To further characterize the toxicity of the formulations, LDH leakage was detected after the administration of all the formulations. It increased compared to the K-R buffer solution-treated group, but it exhibited no significant difference among the groups treated (Table 2). In contrast, LDH leakage was significantly increased by the treatment of sodium taurodeoxycholate, the positive control, compared with all of the formulations (*p* < 0.01).

**Table 2:** Lactate dehydrogenase leakage in the intestinal lumen following pretreatment with various formulations (mean ± SD, *N* = 3).

Formulations	LDH Leakage (U/mL)
K-R buffer solution	2.82 ± 0.13 **
R8	3.05 ± 0.15
R6EW	3.12 ± 0.11
C18R6EW	3.19 ± 0.16
Sodium taurodeoxycholate	3.81 ± 0.10 **

** *p* < 0.01 compared with R8.

R8 were generally reported to be non-toxic and not to induce the leakage of lactate dehydrogenase from the intestinal epithelium at therapeutic concentration [16,33]. The results from the cytotoxicity study suggested that undesirable toxic effects on the intestinal mucosa were not caused by administration of SAR6EW. The results of the LDH leakage study suggested that SAR6EW, R8 and R6EW had no significant differences. These results suggest that SAR6EW has great potential as a powerful tool for insulin delivery across intestinal absorption barriers.

## 3. Experimental Section

### 3.1. Materials

Insulin (28 IU/mg) was purchased from Xuzhou Wanbang Biochemical Pharmaceutical Co., Ltd. (Xuzhou, China). R8 and its derivatives (R8, R6EW, SAR6EW, F-R8, F-R6EW and F-SAR6EW) were purchased from Shanghai Gil Chemical Co., Ltd. (Shanghai, China). All other solvents were of analytical or chromatographic grades and commercially available.

### 3.2. Preparation of R8-Insulin, R6EW-Insulin and SAR6EW-Insulin Samples

The solutions were prepared according to Zhang *et al.* [34] with minor modifications. The SAR6EW was first dissolved by dimethyl sulfoxide (DMSO), then the complex was prepared*.* Specific amounts of insulin were dissolved in 50 μL of 0.1 N HCl in polypropylene tubes, and the insulin solution was diluted with 2.0 mL of phosphate-buffered saline (PBS), pH 7.4, and then normalized with 50 μL of 0.1 N NaOH. The appropriate amount of R8, R6EW and SAR6EW were then measured in a polypropylene tube, and an aliquot of insulin solution was added to the tubes and mixed gently to yield a clear solution. The solution contained a 1:1 molar ratio of insulin and CPP. The three solutions were made, then filtered through a 0.22 μm PTFE filter. The filtrates were frozen and lyophilized for 12 h.

### 3.3. Circular Dichroism

Circular dichroism (CD) was carried out according to Pourhosseini *et al.* [35] with minor modifications. CD spectra were recorded on an Aviv 215 CD spectrophotometer (Aviv Biomedical Inc., Lakewood, NJ, USA) at 25 °C. Thermal unfolding curves were obtained at 222 and 217 nm by increasing the temperature from 30 to 100 °C at a heating rate of 2 °C/min. The scans were obtained for buffer contributions.

### 3.4. Isothermal Titration Calorimetry (ITC)

ITC measurements were carried out at 25 °C on a TA instruments Nano-ITC (TA Instruments, New Castle, DE, USA). All solutions were degassed before using by stirring under vacuum. The sample cell was loaded with insulin solution, but the reference cell contained PBS. The solution in the cell was stirred at a rate of 307 rpm with a syringe (equipped with a micro-propeller) and filled with R8 derivatives solution to ensure rapid mixing. After baseline stability had been achieved, injections were started. The titration involved 30 consecutive injections of the ligand solution, the first being 3 μL, and the remaining ones of 10 μL. In all cases, each injection was done in 6 s at 3 min intervals. In order to correct for the thermal effects, control experiments were done where identical aliquots were injected into the buffer solution. The data were collected automatically and fitted to a one-site binding model by the manufacturer. The first injection was not taken into consideration for data analysis. After subtracting the heat of dilution, a non-linear least-squares algorithm along with the concentrations of the titrant and the sample were used to fit the heat flow per injection to an equilibrium binding equation, providing best fit for the change in enthalpy (ΔH°), the values of stoichiometry (n), and the binding constant (K).

### 3.5. Enzymatic Stability Study

To assess the protective effect against gastrointestinal degradation, the R8-insulin, R6EW-insulin, and SAR6EW-insulin were incubated with a simulated intestinal fluid (pH = 6.8 with α-chymotrypsin or trypsin) in a water bath oscillator at 37 °C. At pre-determined time intervals, samples were withdrawn and the enzymatic reaction stopped by adding 50 µL of ice-cold 0.5% TFA. The remaining amount of insulin was assayed with HPLC. All the experiments were performed in hexaplicate.

### 3.6. In Vitro Cellular Studies

#### 3.6.1. Caco-2 Cell Culture

Caco-2 cells were grown in an incubator at 37 °C under 5% CO_2_. Cells were cultured in T-75 flasks using Modified Eagle’s Medium (MEM) which were supplemented with 20% fetal bovineserum, 1% non-essential amino acids, 10,000 U/mL penicillin and 10,000 μg/mL streptomycin. Cells were passaged at 80%–90% confluency using 0.25% trypsin/ethylenediamine tetra acetic acid solution.

#### 3.6.2. Transport of Complexes across a Caco-2 Cell Monolayer

The Caco-2 cell monolayer on the membrane filter was placed between the donor and receptor compartments and used as a membrane. We then carried out the permeability test (Peff) according to Zhang *et al.* [34].

#### 3.6.3. Cellular Internalization Mechanism

Cellular uptake was performed with specific endocytosis inhibitors or at various temperatures. Caco-2 cells were cultured as described above. In the specific inhibition studies, the cells were pre-incubated with different endocytosis inhibitors including chlorpromazine (CPZ, 10 μg/mL), sodium azide (NaN_3_, 1 mg/mL), methyl-β-cyclodextrin (MβCD, 10 mg/mL), and 5-(*N*-ethyl-*N*-isopropyl) amiloride (EIPA, 10 μg/mL) in HBSS for 1 h at 37 °C. In the temperature dependent uptake studies, the cells were incubated with test solution at 4 °C. The cellular uptake of FITC-insulin was investigated by using confocal laser scanning microscopy (CLSM) and flow cytometry.

### 3.7. In Vivo Studies: Effectiveness of Insulin-CPP Complexes

Male Wistar rats were fed from weaning either an LSD (0.06% Na, TD 92121-Harlan Teklad, Indianapolis, IN, USA), NSD (0.5% Na, TD92140). Rats which had 250–300 g, blood glucose levels >16 mmol/L were chosen. Then the rats were fastened overnight, but had free access to water. The diabetic rats were divided into six groups, each with a minimum of 5 rats. The first group was the diabetic control. Four groups received insulin, R8-insulin, R6EW-insulin, or SAR6EW-insulin formulations at a dose of 25 IU/kg animal body weight. Another group of rats underwent the same surgery and was given 1 IU/kg of insulin solution by subcutaneous injection. The test formulations were placed in enteric-coated capsules (purchased from Qiangji Pharmaceutical Factory, Guangzhou, China, exclusively for small animals), and then administered orally. All the experimental treatments were in agreement with the National Institute of Health’s Guide for the Care and Use of Laboratory Animals.

### 3.8. Cytotoxicity of the R8 Derivatives

#### 3.8.1. Cytotoxicity in Caco-2 Cells

Cytotoxicity was studied in Caco-2 cells according to the method presented by Sajeesh *et al.* [36]. Caco-2 cells used for cytotoxicity assay were seeded into 96-well culture plates at a seeding density of 2 × 10^4^ cells/well. Once a monolayer was obtained, culture medium was replaced with R8 derivatives solution in PBS and cells were incubated at 37 °C for 4 h. Data are expressed as the percentage of viable control cells calculated from the absorbance at 570 nm by comparing the test with control systems.

#### 3.8.2. Lactate Dehydrogenase (LDH) Leakage

LDH leakage from the intestinal membrane was measured according to Liu *et al.* [37]. Dispersions of 1 mL R8 derivatives (5 mg/mL) were administered to the ileum and left in the ileal segments for 2 h. An aliquot of 1 mL Kreb–Ringers (K–R) buffer solution and 1% (*w*/*v*) sodium taurodeoxycholate was also administered to the ileum as the negative and positive controls, respectively.

### 3.9. Statistical Analysis

All experiments were repeated at least three times. Results were expressed as mean values and standard error (mean ± SE). Significance differences between the treatments and the respective controls were determined based on Student’s t test. A *p* value of <0.05 was considered to be significant.

## 4. Conclusions

The results obtained in this study suggest the potential of stearic acid modification of arginine-rich CPPs to improve insulin delivery efficacy. We employed R8 as a typical arginine-rich CPP, and systematically changed its structure by substituting with different aminos, then modifying with stearic acid. It was shown to produce a considerable improvement in the absorption and no sign of toxicity was observed. Due to the existence of stronger interaction between insulin and SAR6EW, higher enzymatic stability was observed for SAR6EW-insulin. Greatly improved cellular uptake, and altered internalization pathways were also observed for SAR6EW-insulin. The improved absorption could be the comprehensive result of multiple factors that might affect each other. Moreover, the concrete mechanism needs further study. Taken together, the results demonstrate that SAR6EW is a promising epithelium permeation enhancer for insulin and suggest that the chemical modification of cell-penetrating peptides is a feasible strategy to enhance their potential.
